# Performance and quality attributes of okra (*Abelmoschus esculentus* (L.) Moench) fruits grown under soil applied Zn-fertilizer, green biomass and poultry manure

**DOI:** 10.1038/s41598-021-87663-4

**Published:** 2021-04-15

**Authors:** Christopher Muyiwa Aboyeji, Samuel Olatunde Dahunsi, Deborah Oluwatosin Olaniyan, Oluwagbenga Dunsin, Aruna Olasekan Adekiya, Adeniyi Olayanju

**Affiliations:** 1 College of Agricultural Sciences, Landmark University, Omu-Aran, Kwara State Nigeria; 2 Microbiology Programme, College of Agriculture, Engineering and Science, Bowen University, Iwo, Osun State Nigeria; 3 Department of Agricultural and Biosystems Engineering, Landmark University, Omu-Aran, Kwara State Nigeria; 4 Landmark University SDG 1 (No Poverty Group), Omu-Aran, Kwara State Nigeria; 5 Landmark University SDG 2 (Zero Hunger Group), Omu-Aran, Kwara State Nigeria; 6 Landmark University SDG 3 (Good Health and Well-Being Group), Omu-Aran, Kwara State Nigeria; 7 Landmark University SDG 9 (Industry, Innovation and Infrastructure Group), Omu-Aran, Kwara State Nigeria; 8 Landmark University SDG 12 (Responsible Consumption and Production Group), Omu-Aran, Kwara State Nigeria; 9 Landmark University SDG 15 (Life on Land Group), Omu-Aran, Kwara State Nigeria

**Keywords:** Plant sciences, Health care

## Abstract

Field experiments were carried out in 2018 and 2019 cropping seasons at Landmark University Teaching and Research farm, Omu-Aran Kwara state, Nigeria, to determine the effect of soil applied Zn-fertilizer, *Tithonia diversifolia* (Ti), *Chromolaena odorata* (Ch) and poultry manure (PM) on the performance, yield, minerals and vitamins composition of okra fruits. Treatments were combined and tested as follows:—Control (T_1_), Control + 10 kg ha^−1^ Zn (T_2_), 5 t ha^−1^ Ti + 5 t ha^−1^ PM (T_3_), 5 t ha^−1^ Ch + 5 t ha^−1^ PM (T_4_), 5 t ha^−1^ Ch + 5 t ha^−1^ Ti (T_5_), 10 t ha^−1^ Ti + 0 kg ha^−1^ Zn (T_6_), 10 t ha^−1^ Ti + 10 kg ha^−1^ Zn (T_7_), 10 t ha^−1^ Ch + 0 kg ha^−1^ Zn (T_8_), 10 t ha^−1^ Ch + 10 kg ha^−1^ Zn (T_9_), 10 t ha^−1^ PM + 0 kg ha^−1^ Zn (T_10_), and 10 t ha^−1^ PM + 10 kg ha^−1^ Zn (T_11_). The experiment was laid out in a randomized complete block design with four repetitions. Vegetative, yield and quality parameters of okra were taken. Data were subjected to analysis of variance and means were compared using Duncan Multiple Range Test (DMRT) at p ≤ 0.05. Variations were observed on the vegetative parameters, yield, minerals and vitamin composition of okra among the applied amendments. The combined application of green biomass, poultry manure, and Zn-fertilizer improved all the variables tested as compared to when they were applied singly. Application of Zn-fertilizer to some selected plots significantly increased yield, Zn, Mg and vitamins concentration of okra. Application of 5 t ha^−1^ Ti + 5 t ha^−1^ PM + 10 kg ha^−1^ Zn (T_3_) and 5 t ha^−1^ Ch + 5 t ha^−1^ PM + 10 kg ha^−1^ Zn (T_4_) significantly improved all the parameters tested but the use of 5 t ha^−1^ Ti + 5 t ha^−1^ PM + 10 kg ha^−1^ Zn (T_3_) resulted in optimum yield and at the same time increase minerals and vitamin concentration of okra. The results of this study therefore showed that *Tithonia diversifolia* (Ti) as green biomass contained and released more and quality nutrients than *Chromolaena odorata* (Ch) when combined with equal rate of Zn fertilizer and poultry manure.

## Introduction

Vegetable crops play an important role in human nutrition and health management in which they are reliable sources of vitamins, minerals and income^1,2^. Fresh fruit is a good source of vitamins, minerals, and plant protein^3^. Rehn and Espig^4^ stated that okra contains about 20% edible oil and protein, while its mucilage is utilized for medicinal purposes. Okra fruit is a traditional valuable nutrient vegetable that provides a good source of vitamin C and other vitamins such as vitamin A, B6, K, pyridoxine, and folates, it contains minerals such as calcium, iron, phosphorus that help in regulating the physiological activities in the body.

Okra has a huge potential for enhancing livelihoods in urban and rural areas^5^ and it is also regarded as the powerhouse of valuable nutrients that is low in calories and is fat-free^6^. Okra fruit also provides significant antioxidant properties, mostly due to their high content in vitamin C, carotenoids, and flavonoids^7^, as well as therapeutic properties against diabetes, hyperlipidemia, microbes, ulcers and neurodegenerative diseases^8^.

Green biomass^9^ and poultry manure^10^ are both organic materials that have been discovered to contain high mineral content such as nitrogen, phosphorus, potassium, calcium, and magnesium and some micronutrients that are capable of improving yields and quality of vegetable crops. Organic manures are also known to be environmentally friendly and have been found to be a good source of nutrients in rejuvenating poor soils by improving crop quality, organic matter and the physical and chemical properties of the soil^11^.

Zinc is a micronutrient that has a great influence on the growth, yield and quality of vegetable crops. Zinc is a constituent element that accelerates the performance of many plant enzymes and chlorophyll synthesis. The mechanism of action of carbonic anhydrase and a number of dehydrogenases which are some of the important enzymes in plants are activated by the zinc element^12^. Deficiency of zinc restrain protein integration as a result of restriction of RNA synthesis^13^. Tryptophan, a precursor of auxin which is accelerated by the application of zinc, is important in flower bud formation, fruit setting and fruit yield^14^. Uptake of soil nitrogen and potash is also known to be increased by the application of zinc. Presence of zinc in the soil avert excessive absorption of P by the roots which may be transported to the leaves^15^. Deficiency of zinc produces changes in leaf morphology and cell histology, which causes several-known disorders “little leaf” or “rosette mottled leaf” etc. its deficiency also causes interveinal chlorosis, reduce root growth, blossoming and fruiting.

Similarly, shortened internodes and chlorotic areas on the older leaves and yellowing of younger leaves due to its deficiency were reported by^16^.

*Chromolaena odorata* originated from North America and belongs to the Asteraceae family. *C. odorata* is very common in the southwest Nigeria where it grows in abundance and restore soil fertility^17^. *Tithonia diversifolia*, a shrub also of the Asteraceae family which originated from Mexico and Central America can now be found in the inter-tropical belts of Asia and Africa^18^. The high quantity of some macronutrients (N, P and K) present in *T. diversifolia* and its ability to extract some other nutrient needed for plant growth in the soil has evoked many research interest^19^. Gachengo et al.^20^ noted that high amount of N element in *T. diversifolia* biomass facilitated its mineralization thereby making N readily available to crops. In Kenya^19^, Malawi^21^, Rwanda^22^ and Zimbabwe^23^, leaves of *T. diversifolia* has been found to improve soil fertility and structure leading to increased crop yield.

Despite the nutritional quality of okra, there are few published articles about using organic materials^24^ and Zn-based fertilizer^25^ in improving the quality of crop. The study was therefore carried out to determine the performance of *T. diversifolia and C. odorata* (green biomass) when combined with Zn fertilizer and poultry manure on the yield and nutritional quality of okra fruits.

## Results

### Initial soil properties

The pre-planting soil analysis is as shown in Table 1. The pH of the soil was strongly acidic, the available phosphorus and nitrogen were relatively low, and the exchangeable K, Ca, and Mg was moderate. The organic matter was adequate. There was a high quantity of sand, with a relatively low quantity of silt and clay in the soil, therefore, the textural class sandy loam.

**Table 1:** Soil physical and chemical characteristics of the experimental sites at 0 -15 cm layer.

Attributes	Values	
	2018	2019
Sand (%)	76	78
Silt (%)	13	12
Clay (%)	11	10
Textural class	Sandy loam	Sandy loam
pH (H_2_O)	5.25	5.23
Total nitrogen (%)	0.16	0.14
Organic matter (%)	3.24	3.15
**Exchangeable bases**		
k (cmol kg^−1^)	0.14	0.10
Na (cmol kg^−1^)	0.66	0.63
Mg (cmol kg^−1^)	0.36	0.32
Al + H (cmol kg^−1^)	1.32	1.29
ECEC (cmol kg^−1^)	0.07	0.05
Available phosphorus (mg kg^−1^)	9.50	8.97
Zn (mg kg^−1^)	0.45	0.43

### Chemical composition of amendments

Table 2 shows the chemical composition of the organic materials used as amendment. Poultry manure had a lower C: N ratio but higher nitrogen, phosphorus, magnesium, organic matter, and organic carbon concentration.

**Table 2:** Chemical composition of the amendments used.

Amendment (%)	O.M (%)	O.C (%)	N (%)	P (%)	K (%)	Ca (%)	Mg (%)	Zn (%)	Fe (%)	Cu (%)	C:N
Poultry manure	37.25	21.61	2.88	1.30	1.67	0.89	0.54	0.23	0.105	0.35	7.50
Tithonia	25.50	14.79	1.88	0.79	3.89	3.41	0.08	ND	ND	ND	7.80
Chromolaena	26.89	15.60	1.21	0.61	1.03	2.30	0.04	ND	ND	ND	12.90

*O.M* organic matter, *O.C* organic carbon, *ND* not detected.

### Effects of soil-applied Zn-fertilizer, green biomass, and poultry manure on the vegetative parameters of okra

Vegetative variables of okra were influenced by various types and doses of soil amendments (Table 3). In 2018 and 2019, when compared with the control (T_1_) which gave statistically lower values for vegetative variables but similar to the application of the control + 10 kg ha^−1^ Zn (T_2_), plots treated with 5 t ha^−1^ Ti + 5 t ha^−1^ PM + 10 kg ha^−1^ Zn (T_3_) produced higher values for plant height, the number of leaves and stem girth though the values were not significant with the application of 5 t ha^−1^ Ch + 5 t ha^−1^ PM + 10 kg ha^−1^ Zn (T_4_). Plots treated with 10 t ha^−1^ PM + 0 kg ha^−1^ Zn (T_10_) and 10 t ha^−1^ PM + 10 kg ha^−1^ Zn (T_11_) resulted in statistically higher values when compared with 5 t ha^−1^ Ch + 5 t ha^−1^ Ti + 10 kg ha^−1^ Zn (T_5_), 10 t ha^−1^ Ti + 0 kg ha^−1^ Zn (T_6_), 10 t ha^−1^ Ti + 10 kg ha^−1^ Zn (T_7_), 10 t ha^−1^ Ch + 0 kg ha^−1^ Zn (T_8_) and 10 t ha^−1^ Ch + 10 kg ha^−1^ Zn (T_9_).

**Table 3:** Effects of soil applied Zn-fertilizer, green biomass and poultry manure on vegetative parameters of okra at 7 weeks after sowing (WAS) in 2018 and 2019 cropping seasons.

Treatments	2018			2019		
	Plant height (cm)	Number of leaves	Stem girth (cm)	Plant height (cm)	Number of leaves	Stem girth (cm)
Control (T_1_)	15.08d	10d	7.40c	13.22c	10d	6.70c
Control + 10 kg ha^−1^ Zn (T_2_)	17.25d	11d	7.90c	14.55c	12d	7.55c
5 t ha^−1^ Ti + 5 t ha^−1^ PM + 10 kg ha^−1^ Zn (T_3_)	36.33a	25a	13.71a	34.88a	24a	12.63a
5 t ha^−1^ Ch + 5 t ha^−1^ PM + 10 kg ha^−1^ Zn (T_4_)	35.67a	24a	13.24a	34.62a	23a	12.58a
5 t ha^−1^ Ch + 5 t ha^−1^ Ti + 10 kg ha^−1^ Zn (T_5_)	28.90c	18c	10.90b	27.85b	18c	9.80b
10 t ha^−1^ Ti + 0 kg ha^−1^ Zn (T_6_)	28.58c	17c	10.44b	28.74b	16c	9.68b
10 t ha^−1^ Ti + 10 kg ha^−1^ Zn (T_7_)	28.87c	18c	10.52b	28.78b	17c	9.87b
10 t ha^−1^ Ch + 0 kg ha^−1^ Zn (T_8_)	27.58c	17c	10.76b	27.65b	17c	9.59b
10 t ha^−1^ Ch + 10 kg ha^−1^ Zn (T_9_)	27.33c	18c	10.80b	27.84b	17c	9.88b
10 t ha^−1^ PM + 0 kg ha^−1^ Zn (T_10_)	31.50b	20b	12.46a	32.00a	20b	11.52a
10 t ha^−1^ PM + 10 kg ha^−1^ Zn (T_11_)	32.17b	21b	12.61a	32.55a	21b	11.65a
Standard deviation	6.65	4.61	2.00	7.25	4.24	1.88

Means in a column under any given treatment followed by the same letter do not differ significantly at p ≤ 0.05 by Duncan multiple range test.

*Ti* tithonia, *Ch* chromolaena, *PM* poultry manure.

### Effects of soil-applied Zn-fertilizer, green biomass, and poultry manure on the yield of okra

Data on the yield of okra as influenced by soil-applied Zn-fertilizer, green biomass and poultry manure is as shown in Table 4. There was a significant response of okra yield to different types and rates of soil amendments. The two years study showed that number of fruits were significantly higher with plots treated with 5 t ha^−1^ Ti + 5 t ha^−1^ PM + 10 kg ha^−1^ Zn (T_3_), 5 t ha^−1^ Ch + 5 t ha^−1^ PM + 10 kg ha^−1^ Zn (T_4_), 10 t ha^−1^ PM + 0 kg ha^−1^ Zn (T_10_) and 10 t ha^−1^ PM + 10 kg ha^−1^ Zn (T_11_). Other treatments gave similar but varying yield values which were statistically higher than the control (T_1_) and control + 10 kg ha^−1^ Zn (T_2_).

**Table 4:** Effects of soil applied Zn-fertilizer, green biomass and poultry manure on yield of okra in 2018 and 2019 cropping seasons.

Treatments	2018			2019		
	Number of fruits per Plot	Number of fruits per hectare	Fruit yield (kg ha^−1^)	Number of fruits per plot	Number of fruits per hectare	Fruit yield (kg ha^−1^)
Control (T_1_)	25.00c	62,500c	310.38d	23.00d	57,500d	304.55e
Control + 10 kg ha^−1^ Zn (T_2_)	27.00c	67,500c	324.41d	26.00c	65,000c	355.30d
5 t ha^−1^ Ti + 5 t ha^−1^ PM + 10 kg ha^−1^ Zn (T_3_)	47.67a	119,175a	1196.13a	46.54a	116,350a	1118.72a
5 t ha^−1^ Ch + 5 t ha^−1^ PM + 10 kg ha^−1^ Zn (T_4_)	46.00a	115,500a	1160.55a	46.00a	115,000a	1098.75a
5 t ha^−1^ Ch + 5 t ha^−1^ Ti + 10 kg ha^−1^ Zn (T_5_)	41.67b	104,175b	1110.05b	41.25b	103,125b	1038.88b
10 t ha^−1^ Ti + 0 kg ha^−1^ Zn (T_6_)	40.00b	100,000b	994.68c	40.10b	100,250b	983.33c
10 t ha^−1^ Ti + 10 kg ha^−1^ Zn (T_7_)	41.00b	102,500b	1062.33b	40.75b	101,875b	1033.21b
10 t ha^−1^ Ch + 0 kg ha^−1^ Zn (T_8_)	39.67b	99,175b	996.23c	39.15b	97,875b	978.74c
10 t ha^−1^ Ch + 10 kg ha^−1^ Zn (T_9_)	38.33b	95,825b	1065.05b	39.62b	99,050b	1044.55b
10 t ha^−1^ PM + 0 kg ha^−1^ Zn (T_10_)	46.66a	116,650a	999.70c	45.15a	112,875a	991.63c
10 t ha^−1^ PM + 10 kg ha^−1^ Zn (T_11_)	47.33a	118,325a	1069.13b	45.85a	114,625a	1060.42b
Standard deviation	7.70	19,300.50	312.30	7.90	19,741.62	290.30

Means in a column under any given treatment followed by the same letter do not differ significantly at p ≤ 0.05 by Duncan multiple range test.

*Ti* tithonia, *Ch* chromolaena, *PM* poultry manure.

### Effects of soil-applied Zn-fertilizer, green biomass, and poultry manure on micronutrients and vitamins composition of okra fruits

The concentration of the micronutrients (Zn, Cu, Fe, and Mg) and vitamins (B6, B9, A, and C) in okra fruits are presented in Table 5. Plots treated with 5 t ha^−1^ Ti + 5 t ha^−1^ PM + 10 kg ha^−1^ Zn (T_3_) significantly increased values for all the micronutrients, though the values were statistically with plots treated with 5 t ha^−1^ Ch + 5 t ha^−1^ PM + 10 kg ha^−1^ Zn (T_4_) and 10 t ha^−1^ PM + 10 kg ha^−1^ Zn (T_11_) except for Cu at 10 t ha^−1^ PM + 10 kg ha^−1^ Zn (T_11_). Compared with the control (T_1_) which gave the least value for Zn and Mg, other plots treated with Zn either sole or in combination with other treatments significantly increased Zn and Mg composition of okra. In a similar vein, application of 5 t ha^−1^ Ch + 5 t ha^−1^ Ti + 10 kg ha^−1^ Zn (T_5_), 10 t ha^−1^ Ti + 0 kg ha^−1^ Zn (T_6_), 10 t ha^−1^ Ti + 10 kg ha^−1^ Zn (T_7_), 10 t ha^−1^ Ch + 0 kg ha^−1^ Zn (T_8_), 10 t ha^−1^ Ch + 10 kg ha^−1^ Zn (T_9_) resulted in similar but varying values for Cu and Fe that are statistically higher than the control plots and plots treated with Zn only.

**Table 5:** Effects of soil applied Zn-fertilizer, green biomass and poultry manure on micronutrients and vitamins concentration of okra fruits (pooled analysis of 2018 and 2019 cropping seasons).

Micronutrients (mg/100 g)					Vitamins (mg/100 g)			
Treatments	Zn	Cu	Fe	Mg	Vit.B6	Vit.B9	Vit.A	Vit.C
Control (T_1_)	0.19e	0.30c	0.43b	21.00d	0.08c	0.20c	0.04d	12.22d
Control + 10 kg ha^−1^ Zn (T_2_)	0.73b	0.33c	0.42b	29.00c	0.11c	0.22c	0.06c	13.15d
5 t ha^−1^ Ti + 5 t ha^−1^ PM + 10 kg ha^−1^ Zn (T_3_)	0.99a	0.55a	0.74a	43.00a	0.23a	0.51a	0.13a	22.33a
5 t ha^−1^ Ch + 5 t ha^−1^ PM + 10 kg ha^−1^ Zn (T_4_)	0.99a	0.55a	0.73a	41.52a	0.21a	0.50a	0.12a	21.70a
5 t ha^−1^ Ch + 5 t ha^−1^ Ti + 10 kg ha^−1^ Zn (T_5_)	0.74b	0.36c	0.45b	42.45a	0.17b	0.45b	0.09b	17.60c
10 t ha^−1^ Ti + 0 kg ha^−1^ Zn (T_6_)	0.43d	0.38c	0.44b	32.54b	0.16b	0.41b	0.06c	17.64c
10 t ha^−1^ Ti + 10 kg ha^−1^ Zn (T_7_)	0.78b	0.33c	0.42b	42.52a	0.17b	0.44b	0.09b	17.52c
10 t ha^−1^ Ch + 0 kg ha^−1^ Zn (T_8_)	0.40d	0.39c	0.43b	35.64b	0.15b	0.42b	0.06c	17.54c
10 t ha^−1^ Ch + 10 kg ha^−1^ Zn (T_9_)	0.78b	0.33c	0.41b	46.46a	0.17b	0.46b	0.09b	17.58c
10 t ha^−1^ PM + 0 kg ha^−1^ Zn (T_10_)	0.52c	0.58b	0.76a	35.44b	0.21a	0.41b	0.13a	19.63b
10 t ha^−1^ PM + 10 kg ha^−1^ Zn (T_11_)	0.98a	0.58b	0.76a	45.43a	0.22a	0.45b	0.14a	19.00b
Standard deviation	0.27	0.17	0.16	7.85	0.05	0.10	0.03	3.06

Means in a column under any given treatment followed by the same letter do not differ significantly at p ≤ 0.05 by Duncan multiple range test.

*Ti* tithonia, *Ch* chromolaena, *PM* poultry manure.

Application of all the amendments either as sole or combined had effects on the availability of vitamins in okra fruits. Control plots gave significantly lower values for all the vitamins though similar to the application of Control + 10 kg ha^−1^ Zn (T_2_) except for vitamin A which was significantly higher than the control. Combination of lower rates of green biomass, poultry manure and Zn enhanced the vitamins composition of okra fruits though the values were only similar when 10 t ha^−1^ PM + 0 kg ha^−1^ Zn (T_10_) and 10 t ha^−1^ PM + 10 kg ha^−1^ Zn (T_11_) were applied except for vitamins B6 and A. Varying but similar values for all vitamins were observed with other treatments except for vitamin A where addition of Zn to the biomass significantly increased its value.

## Discussion

Laboratory analysis of the chemical composition of *T. diversifolia, C. odorata,* and poultry manure used for the study revealed that they all contained macro and micronutrients in varying proportions suitable for the cultivation of okra. The pre-planting analysis of the experimental soil also showed that the nutrient status of the soil was low in major macronutrients. The low fertility status of the soil could be as a result of inherent or continuous cropping on the same piece of land over a period of time without amending the soil with either organic manure or inorganic fertilizer.

That combined application of green biomass with poultry manure increased vegetative parameters could be a result of integrating two or more different sources of nutrients. Adekiya^26^ found that adequate application of poultry manure to mulches should be encouraged in the cultivation of tomatoes, especially where farmers use Acacia legume materials as mulch to maximize their contribution to soil and crop productivity. Nitrogen is an important element in plant growth and development. It plays a key role in chlorophyll, protein, nucleic acid, hormone and vitamin synthesis and also helps in cell division and cell elongation. The positive interaction of the combined mineralization of N present in the amendments could also be an indication of the increase in the vegetative parameters. Higher N mineralization occurs where organic amendments were combined, compared to their individual application, indicating a synergistic relationship between the inputs^27^.

Poultry manure is an effective natural source of nutrients^28^, it is also known to be a better soil amendment compared with some animal manure and chemical fertilizers because of its greater capability to preserve its N^29^. The increase in vegetative parameters of okra could also be a result of the presence of both macro and micronutrients in the PM and their ability to improve the water holding capacity of the soil. Animal manures have been shown to supply required plant nutrients, improve soil structure and water holding capacity, increase microbial population, and promote plant growth^30^. Higher yield was also recorded in all plots containing PM. The superior N supply by poultry manure may have contributed to being the better growth and yield of okra in plots with poultry manure. The results corroborated the finding of^31^ where they observed that poultry manure increased the performance of okra relative to other amendments. The poor vegetative and yield parameters observed at the control and Control + 10 kg ha^−1^ Zn (T_2_) plots might be a result of the low fertility status of the experimental soil as determined by the laboratory analysis carried out before the start of the experiment. Application of organic manure increases organic elements’ availability in soil, thereby improving the nutrient use efficiency (NUE) of crops and alleviating the harmful impact of climate change on crop production^32^.

Zn is an important trace element required for plant growth and development. According to the Food and Agriculture Organization (FAO), about 30% of the cultivable soils of the world contain low levels of plant-available Zn^33^. The increase in the vegetative parameters of okra with the application of Zn-fertilizer could be a result of the role of Zn in photosynthesis by increasing the amount of chlorophyll in the leaves and making Mg available to plant. Aboyeji^34^ concluded in their study that combined application of Zn fertilizer with PM and NPK fertilizer resulted in a significant increase in the vegetative parameters of tomato. Increase in the vegetative performance of okra plant could again be attributed to the effect of application of Zn fertilizer which assisted in the nitrogen use efficiency of the plant. Zinc application also helps in increasing the uptake of nitrogen and potash^35^.

The relative increase in okra yield grown under combined application of green biomass, poultry manure, and Zn-fertilizer could be attributed to the applied amendments which supplied the needed nutrient required by the crop for its metabolic functions or as a result of the complementary roles of organic materials and inorganic Zn-fertilizers in improving crop yield. Zinc might have increased the efficiency of added organic materials in the soil and increase the rate of humification of zinc thereby enhancing the availability of both native and added nutrients in the soil and thus increased the yield of okra. The high yield response of okra could also be ascribed to the effect of Zn in making some nutrients available in the soil. Gurmani et al.^36^ found that application of Zn significantly increased the dry biomass, fruit yield, fruit fresh weight, and numbers of fruits per plant in the tomato, the highest increase was found with 10 kg ha^−1^ Zn. The results are also in accordance with the findings of^37^ where they found that the application of zinc in soil increased the availability of zinc in the rhizosphere.

The addition of organic materials to soil leads to a significant increase in macro and micronutrient availability in soil compared to the control^34^. Combined incorporation of green biomass, poultry manure and Zn-fertilizer increased availability of micronutrients in okra fruits than when applied singly. Increased availability of Zn and Mg in okra fruits could be due to the application of Zn which in turn made Mg be available in okra fruits. It could also be attributed to the effect of Zn in increasing the uptake of some nutrients. Singh and Singh^38^ reported that Zn application increased chlorophyll content and raised the concentration of Zn, Ca, Mg, K, and P in tissues.

Results also indicated that combined application of green manure, poultry manure, and Zn-fertilizer increased Fe and Cu concentration of okra fruits similar to the application of sole PM and PM + 10 kg ha^−1^ Zn. This could be attributed to the initially high content of total trace elements in chicken manure. The effect of organic materials on Fe and Cu uptake by okra could be due to the influence of organic carbon which acts as a source of energy for soil microorganisms, which upon mineralization releases organic acids that decreased soil pH and improve the availability of Fe and Cu^39^.

The increased mineral and vitamin contents of okra fruits as a result of combined application of green biomass, poultry manure, and Zn-fertilizer or PM alone can be adduced to increase soil chemical properties which led to greater metabolic activities and hence higher minerals and vitamins in 5 t ha^−1^ Ti + 5 t ha^−1^ PM + 10 kg ha^−1^ Zn (T_3_) and 5 t ha^−1^ Ch + 5 t ha^−1^ PM + 10 kg ha^−1^ Zn (T_4_) plots compared with their sole forms.

Different organic wastes influence the nutritional quality of crops differently. The study revealed that the application of 5 t ha^−1^ Ti + 5 t ha^−1^ PM + 10 kg ha^−1^ Zn (T_3_) and 5 t ha^−1^ Ch + 5 t ha^−1^ PM + 10 kg ha^−1^ Zn (T_4_) significantly improved the minerals and vitamin composition of okra. An increase in the nutritional quality of okra could be attributed to the combined application of green biomass and poultry manure. Integrated application of green biomass, poultry manure, and Zn-fertilizer not only influenced the micronutrient composition of okra but also helped in enhancing its vitamin contents.

Wolf and Snyder^40^ reported that C: N ratio of organic materials markedly influences the decomposition rate and the mineralization of N because N determines the growth and turnover of the microorganisms that mineralize organic carbon. The result also revealed that *T. diversifolia* and PM contained higher N values and lower C: N ratio than *C. odorata*. This could be attributed to the faster mineralization and nutrient release thereby leading to a consistent increase in the agronomic and quality parameters of okra^41^. In their experiments found that the rate of litter decomposition depends on the C: N ratio and/or nitrogen content of the leaf litter.

## Conclusion

Application of 5 t ha^−1^ Ti + 5 t ha^−1^ PM + 10 kg ha^−1^ Zn (T_3_) and 5 t ha^−1^ Ch + 5 t ha^−1^ PM + 10 kg ha^−1^ Zn (T_4_) significantly improved all the variables tested though having similar values with 10 t ha^−1^ PM + 0 kg ha^−1^ Zn (T_10_) and 10 t ha^−1^ PM + 10 kg ha^−1^ Zn (T_11_) in some cases. *T diversifolia* contained higher values for N, P, K, Ca. and Mg with lower C: N which may have facilitated the rate of decomposition and release of nutrients than *C. odorata*, then the optimum dose for better productivity and crop quality in the study area is 5 t ha^−1^ Ti + 5 t ha^−1^ PM + 10 kg ha^−1^ Zn (T_3_).

## Materials and methods

### Description of the experimental site

The experiment was conducted at the Teaching and Research farm of Landmark University, Omu-Aran Kwara State (latitude 8^o^8**′**N and longitude 5^o^6**′**E) with an altitude of 495 m elevation above the sea level in the derived savannah zone of Nigeria. It has an annual rainfall ranging from 600 mm to 1, 200 mm and an annual average temperature of 24.9 °C. The months of December and January coincide with the cold and dry harmattan period. The study area is classified as Aw; which means tropical wet and dry or savanna climate; with the driest month having precipitation less than 60 mm (2.4 inches) and less than 4% of the total annual precipitation.

### Soil sampling and analysis and laboratory determination of poultry manure, *T. diversifolia* and* odorata*

Soil samples were randomly taken from each plot before the commencement of the field experiment. All samples were bulked together to obtain the composite samples for laboratory analysis**.** Poultry manure used was analysed for nutrient composition after being air-dried using warm air (18–21 °C) in the poultry house for 7 days. The dried poultry manure was crushed and passed through a 2-mm sieve. The analysis was done for organic carbon (OC), total N, P, K, Ca, Mg, Cu, Mn, Zn, and Na^42^. Leaf samples of *T. diversifolia* and *C. odorata* were collected fresh, oven-dried for 24 h at 80 °C and grinded in a Willey mill. N was determined by the micro-Kjeldahl digestion method. Ground samples were digested with the nitric-perchloric-sulphuric acid mixture for the determination of P, K, Ca and Mg. Phosphorus was determined colorimetrically using the vanadomolybdate method, K was determined using a flame photometer and Ca and Mg were determined by the EDTA titration method^42^. The percentage of organic carbon in the sample was determined by the Walkley and Black procedure using the dichromate wet oxidation method^43^. Sample pH was determined by using a soil–water medium at a ratio of 1:2 using Jenway digital electronic pH meter model 3520^44^. Contents of Zn, Cu, Mg, and Fe, were determined by an atomic absorption spectrophotometer.

### Treatment and experimental design

Treatments consists of *Tithonia diversifolia* leaves (Ti), *Chromolaena odorata* leaves (Ch), poultry manure (PM) and zinc sulphate (Zn). Treatments were combined and tested as follows:—Control (T_1_), Control + 10 kg ha^−1^ Zn (T_2_), 5 t ha^−1^ Ti + 5 t ha^−1^ PM (T_3_), 5 t ha^−1^ Ch + 5 t ha^−1^ PM (T_4_), 5 t ha^−1^ Ch + 5 t ha^−1^ Ti (T_5_), 10 t ha^−1^ Ti + 0 kg ha^−1^ Zn (T_6_), 10 t ha^−1^ Ti + 10 kg ha^−1^ Zn (T_7_), 10 t ha^−1^ Ch + 0 kg ha^−1^ Zn (T_8_), 10 t ha^−1^ Ch + 10 kg ha^−1^ Zn (T_9_), 10 t ha^−1^ PM + 0 kg ha^−1^ Zn (T_10_) and 10 t ha^−1^ PM + 10 kg ha^−1^ Zn (T_11_). The experiment was laid out in a Randomized Complete Block Design (RCBD) replicated four times. Vegetative, yield and quality parameters of okra were taken.

### Sources of materials

Fresh and tender leaves of *T. diversifolia* and *C. odorata* were collected from a fallow field of Landmark University Teaching and Research farm. Poultry manure was collected from the poultry house of Landmark University farms while zinc sulphate fertilizer was purchased from a reputable agro-allied farm store.

### Land preparation and plot size

The land was ploughed and harrowed to pulverize and loosen the soil. Thereafter the field layout was carried out to mark out the appropriate number of treatment plots. The size of each experimental plot was 2 m × 2 m = 4 m^2^ and there were 11 plots per replicate (4 m × 11 m = 44 m^2^) which were replicated four times. The size of the whole experimental plot was 44 m^2^ × 4 m^2^ = 176 m^2^.

### Seed variety and sowing

The variety used for the experiment was the local variety that is commonly grown and eaten in Omu-Aran, Kwara State, Nigeria. Okra seeds were subjected to a viability test before sowing. The seeds were poured inside a bowl containing clean water and were allowed to stay for 2 min. The floated seeds were discarded while the ones that sank were sown. The seeds were sowed two weeks after the incorporation of the green biomass and poultry manure. The sowing spacing was 60 cm by 30 cm inter and intra row respectively. Two seeds were sown per hole and were later thinned to one plant per stand two weeks after sowing.

### Application of organic materials and Zn-fertilizer

Fresh and tender leaves of both *T. diversifolia* and *C. odorata* were chopped into pieces and incorporated into the soil immediately after land preparation and field layout and were allowed to decompose for two weeks before sowing. Zn-fertilizer was applied to designated plots 2 weeks after sowing by side placement 8 cm away from the base of the plant.

### Weed control

Weed control was manually done using the local hand-held hoe and rouging. Weed control started 2 weeks after germination.

### Harvesting

Mature okra pods were harvested at three days’ intervals, counted and weighed using a weighing balance based on each treatment.

### Data collection

The following parameters were taken during the experiment:—plant height, number of leaves, stem girth, yield per plot/hectare, micronutrients and vitamins composition of okra fruits.

### Laboratory determination of micronutrients (minerals) in okra fruits

Representative okra fruit samples were taken per plot and per replicate to analyze for the levels of mineral elements contained in the fruit at the crop and soil laboratory of Landmark University, Omu-Aran, Nigeria. Mature fresh okra fruits were collected, oven-dried for 24 h at 80 °C, and ground in a Willey mill. Mineral elements were determined according to the methods as recommended by the Association of Official Analytical Chemists^45^. One gram of each sample was digested using 12 cm^−3^ of the mix of HNO_3_, H_2_SO_4_, and HClO_4_ (7:2:1 v/v/v). Contents of Zn, Cu, Mg, and Fe, were determined by an atomic absorption spectrophotometer.

### Laboratory analysis of vitamins in okra fruits

The concentrations of the vitamins (vitamins B6, B9, A, and C) in water extracts of okra samples were determined as previously described by^46^. All analyses were carried out in triplicate.

### Statistical analysis

Data collected were subjected to analyses of variance using Statistical Analysis Software^47^ (SAS, Inc. 2002). The treatment means were compared using the Duncan Multiple Range Test at p ≤ 0.05.

### Ethical declaration

I confirm that all the research meets ethical guidelines and adheres to the legal requirements of the study country.
